# Polyvinyl Butyral (PVB) Nanofiber/Nanoparticle-Covered Yarns for Antibacterial Textile Surfaces

**DOI:** 10.3390/ijms20174317

**Published:** 2019-09-03

**Authors:** Fatma Yalcinkaya, Michal Komarek

**Affiliations:** 1 Department of Nanotechnology and Informatics, Institute of Nanomaterials, Advanced Technologies and Innovation, Technical University of Liberec, Studentska 1402/2, 46117 Liberec, Czech Republic; 2 Institute for New Technologies and Applied Informatics, Faculty of Mechatronics, Technical University of Liberec, Studentska 1402/2, 46117 Liberec, Czech Republic

**Keywords:** PVB, nanoyarn, antibacterial, vanadium, copper, electrospinning

## Abstract

In this study, nanoparticle-incorporated nanofiber-covered yarns were prepared using a custom-made needle-free electrospinning system. The ultimate goal of this work was to prepare functional nanofibrous surfaces with antibacterial properties and realize high-speed production. As antibacterial agents, we used various amounts of copper oxide (CuO) and vanadium (V) oxide (V_2_O_5_) nanoparticles (NPs). Three yarn preparation speeds (100 m/min, 150 m/min, and 200 m/min) were used for the nanofiber-covered yarn. The results indicate a relationship between the yarn speed, quantity of NPs, and antibacterial efficiency of the material. We found a higher yarn speed to be associated with a lower reduction in bacteria. NP-loaded nanofiber yarns were proven to have excellent antibacterial properties against Gram-negative *Escherichia coli* (*E. coli*). CuO exhibited a greater inhibition and bactericidal effect against *E. coli* than V_2_O_5_. In brief, the studied samples are good candidates for use in antibacterial textile surface applications, such as wastewater filtration. As greater attention is being drawn to this field, this work provides new insights regarding the antibacterial textile surfaces of nanofiber-covered yarns.

## 1. Introduction

Nanofibers have attracted growing attention due to their high surface area, highly porous structure, narrow pore size, and low density. The superior properties of nanofibers enable their use in various fields, such as wastewater filtration [1,2], distillation [3,4], desalination [5,6], air filtration [7,8], biomedical application [9,10], gas sensors [11,12], batteries [13,14], data storage [15], and solar cells [16]. Apart from their many advantages, the weak mechanical properties of nanofibers restrict their use in a wider variety of applications. Grothe et al. [17] found that the dimensional stability of nanofibers changes in wet applications. Attempts have been made to improve the mechanical weakness of nanofibers, including polymer blending [18], inorganic blending [19], the use of epoxy composites [20], thermal lamination [21], dip-coating [22], and ultrasonic welding [23].

Recently, considerable effort has been expended to obtain mechanically durable nanofiber yarns to use in smart textiles. The techniques that have been used in the preparation of nanofiber yarns include: (1) direct collection by self-bundling [24], (2) deposition onto a non-solvent [25,26,27], and (3) drawing and twisting from the collector material [28,29]. Wang et al. [24] used the self-bundling electrospinning method to produce continuous nanofiber yarns. In this technology, single-needle electrospinning with a grounded needle tip is used to self-bundle nanofibers at the beginning of the electrospinning process, then the self-bundled yarn is pulled back and wound on a grounded rotating collector. Although the resulting yarns are well aligned, the productivity of this method is extremely low. Smit et al. [26] used another technique involving solution spinning onto a non-solvent reservoir collector and then drawing. However, the theoretical production rate is assumed to be 180 m/hr, which is only suitable for lab-scale production. In another work [29], the authors placed a neutral copper funnel between two oppositely charged needles in a needle electrospinning device. Nanofibers were then collected on a copper plate covered with aluminum foil, which was adhered to the face of the copper funnel. With the funnel rotating at approximately 150 rpm, the collected nanofiber bundle was twisted into yarn. These yarns were then collected onto a winder rotating at a rate of approximately 8 mm/min. The authors found the polymer concentration to significantly affect the ability to form nano yarns, and also that the electrospinning parameters were fairly limited. Despite the successful attempts at yarn production, the productivity achieved has never been sufficient for practical application.

The methods reported in the literature have mainly been based on the needle electrospinning system, which offers very low yields of nanofiber and nano yarn. Recently, Shuakat and Lin [30] prepared nanofiber yarns using a combination of both needle and needleless electrospinning. In this method, the authors prepared a highly tenacious yarn at a production rate of 240 m/h and a twist level up to 4700 twists per meter. Pokorny et al. [31] used alternating current (AC) electrospinning based on a needleless spinning electrode to prepare nanofiber yarn at rotation speeds ranging between 5000 and 20,000 rpm. However, fibers produced in this way were highly tortuous (inter-twined) due to the AC, in which positively and negatively charged jet segments are mutually attracted. Using a needle-free electrospinning system can increase the production rate of nanofiber yarn. In this work, we used a needle-free roller electrospinning system to prepare nanoparticle (NP)-incorporated nanofiber-covered yarns at various production rates. The main aim of this work was to prepare antibacterial nanofiber yarns at a high rate of production for possible application in wastewater cleaning. To do so, we used polyvinyl butyral (PVB) nanofibers with two different nanoparticles, i.e., copper oxide (CuO) and vanadium (V) oxide (V_2_O_5_).

PVB has been used extensively in many applications, due to its low cost, flexibility, optical clarity, and good adhesion to many surfaces [32,33]. We selected a PVB polymer to prepare nanofibers due to its non-toxic solvent system, low cost, and properties that make it conducive to electrospinning. We chose CuO and V_2_O_5_ NPs as antibacterial agents for the prepared nanofiber-covered yarn. CuO has been known for centuries as a bacteriostatic inorganic compound. Moreover, the low cost of CuO increases its applicability as an antibacterial material. CuO nanoparticles exhibit long-lasting antibacterial activity and better stability than macro-sized CuO [34]. In addition to its antibacterial activity, CuO is a semiconducting material with optical, magnetic, and electrical properties [35]. V_2_O_5_ NPs have begun to be used in a variety of fields, including gas sensors [36], batteries [37], solar cells [38], microelectronics [39], and optoelectronic devices [40], and have been proven to be bactericidal agents [41].

In this study, we selected Gram-negative *Escherichia coli* (*E. coli*) to test the antibacterial properties of the above materials. *E. coli* is one of the most frequently used bacterial indicators in the measurement of the quality of drinking and untreated water. The presence of *E. coli* bacteria indicates the potential presence of pathogenic microorganisms in natural and treated waters [42]. *E. coli* itself has been determined to be pathogenic, causing either intestinal (e.g., diarrhea) or extraintestinal (e.g., urinary tract) infections [43].

To the best of our knowledge, this study represents the first use of a semi-industrial-scale needle-free electrospinning device to prepare antibacterial nanocomposites with high-speed productivity. In addition, this is the first time the antibacterial efficiencies of CuO and V_2_O_5_ nanoparticles have been compared. We investigated the quantity of NPs and the production speed of the yarn’s antibacterial activity. Unlike earlier results reported in the literature, we compared the antibacterial performances of the CuO and V_2_O_2_ nanoparticles and produced the nanofiber yarns at high speed. In light of the need to improve the functionality of nanofibers, we found NP-loaded nanofiber-covered yarns to show great promise and potential in various applications, such as water filtration.

## 2. Results and Discussion

### 2.1. Surface Morphology

**Figure 1:**
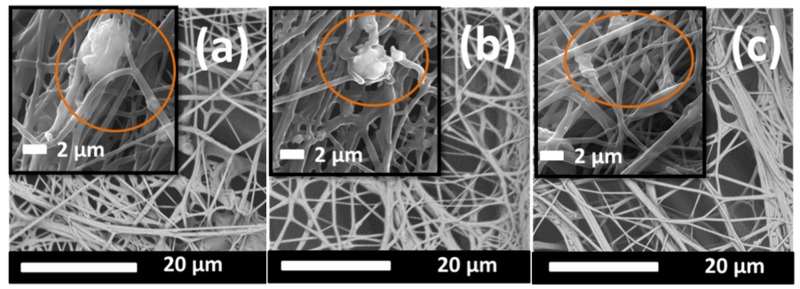
SEM (scanning electron microscopy) images of (**a**) PV_5_100, (**b**) PV_5_150, and (**c**) PV_5_200. Nanoparticles are indicated in the circles.

**Figure 2:**
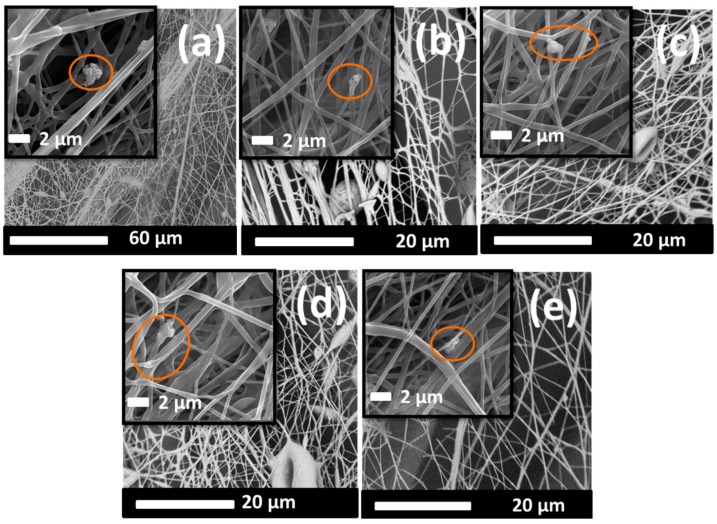
SEM images of (**a**) PC_5_100, (**b**) PC_10_100, (**c**) PC_5_150, (**d**) PC_10_150, and (**e**) PC_5_200. Nanoparticles are indicated in the circles.

Figure 1 and Figure 2 show scanning electron microscopy (SEM) images of the surface morphologies of the fibers.

These SEM images indicate that the speed of the yarn influenced the morphology of the nanofibers, whereby we can see that the lowest speed of the PVB + V_2_O_5_ sample resulted in a bundle structure (Figure 1a). When the speed was increased, fewer fibers were collected, and a less compact structure was formed (Figure 1c). The same result was observed for the PVB + CuO samples (Figure 2a,e). At the highest speed, a more open structure was formed, which indicates the presence of fewer nanofibers and nanoparticles.

The PVB + V_2_O_5_ nanofibers were observed to have a sticky and bundled fiber form. The quality of nanofibers is generally related to their concentration, surface tension, conductivity, and the viscosity of the solution. In this work, we kept the solution concentration of PVB + V_2_O_5_ the same, and changed only the speed of the collector yarn. In previous research, a bundle form for the PVB nanofibers has been observed when both the solution concentration and throughput were high [44]. When the speed of the collector yarn is high, fewer fibers are collected on the surface, so a smaller bundle structure occurs.

Bead structures were observed on the PVB + CuO nanofibers. We suppose that adding CuO increases the conductivity of the PVB solution. As a result, a greater charge can be affected by the electrospinning jet, which can then affect the fiber morphology. A similar result was reported by Demir et al. [45], who found that by increasing the concentration of the magnetic nanoparticles in a poly(e-caprolactone) (PCL) solution from a 1:25 to a 32:25 weight ratio, the fiber diameter changed and bead formation occurred.

**Figure 3:**
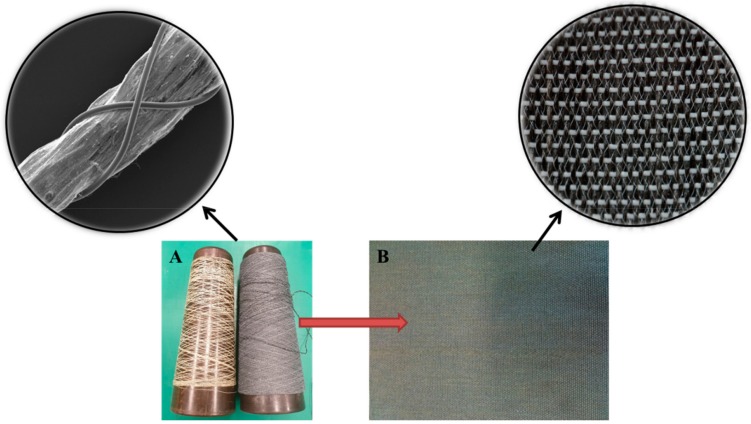
Fabrication of nanofiber-covered yarn: (**A**) nanofiber/NP-covered yarn, (**B**) woven fabric.

Figure 3 shows that the prepared fibers were woven into a textile surface, which means that nanofiber-covered yarn can be used in wearable textiles. Moreover, using different weaving structures (such as a tight weaving structure) enables the use of these surfaces in filtration applications.

### 2.2. Antibacterial Test Result

**Figure 4:**
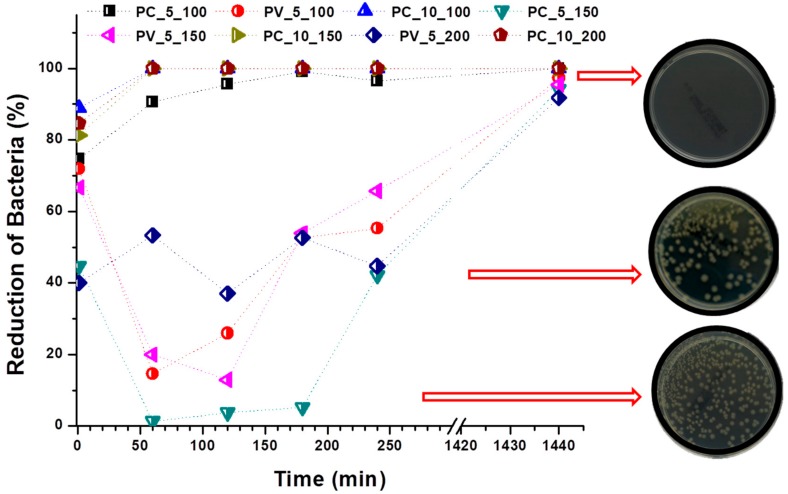
Reduction in bacteria (*Escherichia coli*) over time.

Reproducibility and production speed are two important criteria in the manufacturing of materials. In this work, nanofibers were electrospun under controllable conditions, i.e., temperature and heat. The speed of the collector yarn was controlled using a take-up cylinder, for which the faster the yarn speed, the fewer fibers were collected on the surface of the yarn, as shown in Figure 1 and Figure 2. Table 1 shows the experimental results for the inhibition effect of the yarns against *E. coli* with time. Figure 4 shows the reduction in microorganism with time.

The sizes of bacteria are usually in the micron range for cellular membranes and in the nanometer range for pores. Metal oxides show significant antimicrobial activities when the particle size is reduced to nanometer scale, as nano-sized particles easily interact with bacterial cell surfaces and enter into the cell without any hindrance. The interaction between nanoparticles and bacteria is mostly toxic [46]. As a result, the material shows antibacterial activity. Since the antibacterial activity varies for each nanoparticle, the mechanism of each particle has not yet been fully investigated.

Padmavathy et al. [47] studied the antibacterial activity of zinc oxide (ZnO) with various particle sizes. The authors found that the bacterial efficacy of ZnO nanoparticles increased with decreasing particle size. When the ZnO nanoparticle size decreased, it contributed to severe mechanical damage of the cell membrane and an enhanced bactericidal effect. In another work, the role of the oxidation state in the antibacterial activity of CuO NPs was investigated [48]. The antibacterial activity of CuO is associated with a sudden decrease in cell membrane integrity, the production of reactive oxygen species (ROS), and damage to the genetic material. CuO nanoparticles have been found to produce significant ROS, i.e., superoxides, which promote antibacterial activity.

V_2_O_5_ has shown excellent antibacterial activity against *E. coli* due to the ROS generated within the cells [41]. The toxicity of the vanadium has been found to depend on the amount used [49]. There might be two reasons for the antibacterial activity of V_2_O_5_: (1) the production of ROS and (2) the damaging effect of nanoparticles moving through the cell walls of the bacteria with which they are in contact [50].

At low yarn speed (100 m/min), more nanofibers can be collected on the surface. In previous work, researchers have proven there to be an inverse proportion between the amount of collected PVB yarn and the speed of the yarn [51,52]. Fewer nanofibers means fewer nanoparticles at the surface. Thus, the quantity of NPs/nanofiber on yarn depends on the speed of the yarn.

Figure 4 reveals that sample P0 (without any NPs) had very little effect, almost negligible, on the reduction of bacteria. At zero contact time, the PC_5_150 and PV_5_200 samples showed very low antibacterial effect, with a reduction of less than 50%. The reason for this is the fast speed of the collecting yarn, which resulted in fewer NP/nanofibers forming around the yarn and only a small number of NPs. The 10% CuO NP-loaded PVB nanofibers showed very high antibacterial efficiency for 0–1440 min contact time. The same results were observed for the PC_5_100 sample due to the low speed of the yarn, which led to the presence of more NP/nanofibers around the yarn. In one hour, the antibacterial efficiencies of the PC_10_100, PC_10_150, and PC_10_200 samples were all 99.99%. These results indicate that all samples showed excellent antibacterial activity after 1440 min contact with bacteria. As the number of colonized bacteria was at least 85% lower, we can conclude that the nanoparticles were successful in preventing bacterial adhesion at the surface of the nanofiber-covered yarn. The nanoparticles might interact with the cellular layers of the bacteria and perforate the cell wall, thus realizing antibacterial effects.

In a previous study in our research laboratory [53], we measured the stability of nanoparticles on the surface of a nanofiber web under simulated water-filtration conditions. A constant flow of water (3 L min^−1^ flow rate) was passed through each sample for 8 h (1440 L through each sample). Antibacterial tests (against to *E. coli* and *Staphylococcus gallinarum*) were repeated after the water filtration test to determine the stability of the antibacterial properties and the degree of fixation of the CuO particles onto the structure of the nanofibers. We observed no changes in the antibacterial efficiency.

Previously [51], we studied the antibacterial efficiency of CuO NPs immobilized in composite yarns against Gram-positive *Staphylococcus gallinarum* (*S. gallinarum*) and Gram-negative *E. coli*. The results indicated that the initial antibacterial efficiency of CuO against *S. gallinarum* is higher than that against *E. coli* at “0” contact time. We found CuO nanoparticles to be more effective against Gram-positive microorganisms. After 1 h of contact time, both Gram-positive and Gram-negative bacteria had been disinfected.

According to the strongly supported material characterization results, NP-loaded nanofiber-covered yarns exhibit extremely high antibacterial properties against Gram-negative *E. coli* bacteria. Previously [54], we found the antibacterial efficiency of CuO against *E. coli* to be superior to that of other NPs, such as titanium dioxide (TiO_2_), zinc oxide (ZnO), zirconium dioxide (ZrO_2_), and silver. There have been no reports in the literature comparing the antibacterial efficiencies of CuO and V_2_O_5_ nanoparticles. Based on our overall results (Figure 4 and Table 1) and support from previously reported results, we found CuO to be more effective against Gram-negative *E. coli* than many other NPs, including V_2_O_5_.

## 3. Materials and Methods

We dissolved 11 wt.% PVB, purchased from Kuraray (Mowital B60H, 60 kDa, Hattersheim am Main, Germany), in acetic acid (98%, Penta, s.r.o., Prague, Czech Republic). Then, we added 1 wt.% of the surfactant Triton-X 100 (Sigma Aldrich, Prague, Czech Republic) to the solution to prevent aggregation of the nanoparticles. The solution was then stirred overnight at room temperature. We purchased the copper oxide (CuO) nanoparticles from Penta s.r.o. (Prague, Czech Republic) and the vanadium oxide (V_2_O_5_) from Sigma Aldrich (Prague, Czech Republic). The particle size was not provided. The polymeric solution was mixed with NPs at various concentrations, as shown in Table 2, and dispersed using an ultrasonic disperser for five minutes. In the table, the nanofiber-covered yarn without any nanoparticle additive, indicated as P0, had a core yarn speed of 150 m/min. Since our nanofiber yarns show promise for use in wastewater remediation, we selected the quantity of nanoparticles based on the results of previous research related to nanofiber membranes [51,53]. Both 5 wt.% and 10 wt.% of CuO in nanofiber have been reported to show enormous antibacterial activity against Gram-positive *S. gallinarum* and Gram-negative *E. coli* [53]. In another study, 5 wt.% of V_2_O_5_ nanofiber-incorporated polyethersulfone (PES) was found to remove methylene blue dye pollutant from water [55]. We observed the surface morphologies of the electrospun fibers on an SEM (Vega 3SB, Brno, Czech Republic). We mounted the yarn samples on a stub of metal with double-sided adhesive, coated them with 7 nm of gold, and then made SEM observations at various magnifications and at various places (accelerating voltage 30 kV, beam intensity 7).

### 3.1. Yarn Preparation

**Figure 5:**
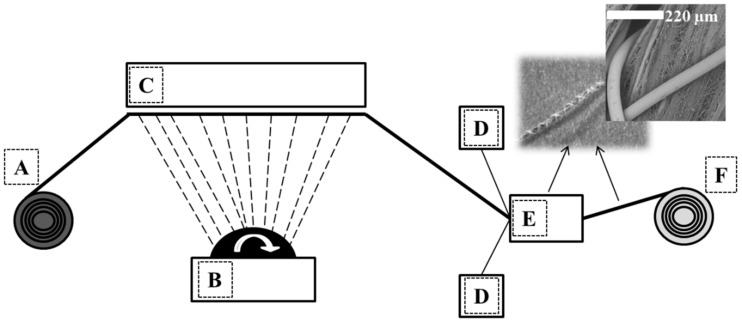
Diagram of a nanofiber-covered-yarn production device. (**A**): Textured core yarn, (**B**): roller electrospinning system, (**C**): collector, (**D**): application of protective yarn(s), (**E**): Twisting of protective yarn, and (**F**): take-up mechanism.

We prepared the NP/nanofiber-covered yarn using a needle-free roller electrospinning system, in which we had modified a rotating roller electrospinning device to enable the covering of yarn with nanoparticle-embossed nanofibers. The core yarn was used as a collector, as shown in Figure 5. The roller electrode was immersed in a polymer solution tank and connected to a high-voltage source (Figure 4). When the electrostatic field overcame the surface tension, fibers formed from the surface of the roller toward the collector yarn. As the core yarn, we used a randomly textured polyester yarn (dtex 167f 36 × 1 × 3). During spinning, the nanofibers surrounded the core yarn, and we withdrew the core yarn at various speeds, i.e., 100 m/min, 150 m/min, and 200 m/min. A faster production speed required a higher speed of the collector yarn, which resulted in fewer nanofibers being collected on the surface. To prevent abrasion of the nanofibers on the surface of the textured yarn, we used a protective yarn to cover the finished yarn. The finished yarns were spooled onto a bobbin and the prepared yarns were transported to the company VÚTS a.s. (Liberec, Czech Republic) to be woven into a plain design.

### 3.2. Antibacterial Test

We purchased Gram-negative *E. coli* bacteria from the Czech Collection of Microorganisms, Masaryk University (Brno, Czech Republic) and incubated the bacteria on sterile agar in a broth agar medium from Brno (Czech Republic). To quantitatively evaluate the antibacterial activity of the samples, we used both the ASTM E 2149-01 method and the AATCC test method 100 (standard test method for determining the antibacterial activity of immobilized antibacterial agents under dynamic contact conditions). The samples were cut to weigh around 1 ± 0.01 g. We sterilized the samples in an oven at 80 °C for 60 min before conducting the tests. We also prepared a blank sample containing no antibacterial agent.

We cultivated the *E. coli* microorganisms in a sterilized LB broth medium and then incubated them overnight at 37 °C in a shaking incubator. The bacterial suspensions included 10^6^ colony forming units (CFU).

After diluting the bacterial suspensions to between 10^2^ and 10^3^ CFU, we placed the sterilized samples into individual sterilized test tubes and inoculated them with 30 mL of the *E. coli* bacterial suspension. At “0” contact time (first minute of contact) and after 1 h, 2 h, 3 h, 4 h, and 24 h, we extracted 600 μL of bacterial suspension and then quickly spread it onto tryptic soy agar plates. We determined the number of viable *E. coli* by plating the extracted solution onto the Tryptic Soy agar plates and counting the colonies after 24 h of incubation at 37 °C [51].

Next, we used the following equation to calculate the reduction in the number of microorganisms in the test tubes with the nanofiber membranes:

$$R=\frac{100\left(A1-A0\right)}{A0},$$

where *R* is the percentage reduction of the test microorganism, *A*1 is the number of bacteria recovered from the inoculated nanofiber-covered yarn with nanoparticles in the test tube after the specified contact time, and *A*0 is the number of bacteria recovered from the inoculated nanofiber-covered yarn with nanoparticles in the test tube at zero contact time.

## 4. Conclusions

In this study, we prepared nanofiber-covered yarn using a needleless electrospinning system. We loaded the nanofiber layer with CuO and V_2_O_5_ nanoparticles to improve the antibacterial efficiency of the prepared yarn. Our experimental results indicate that there is a direct relationship between the yarn preparation speed and the antibacterial efficiency of the yarn. High-speed production yields lower nanofiber coverage on the core yarn, which results in less bacterial reduction. However, the number of NPs in the mixture is more important than the speed of the prepared yarn. Thus, we can conclude that CuO NPs showed better antibacterial efficiency than V_2_O_5_ and NP-loaded nanofiber yarn was successfully produced. Based on the efficient antibacterial activities of CuO and V_2_O_5_ nanoparticles, these yarns hold great promise for the disinfection of microorganisms under practical indoor or outdoor conditions, such as in air and water filters or in textiles. We believe that the preparation of such yarns will promote the development of more active materials for next-generation antibacterial applications.

## Figures and Tables

**Table 1:** Inhibition effect of the nanofiber/NPs-coated yarn in time.

Sample	%Reduction of *E. coli* in Time					
	0 Contact Time	1 h	2 h	3 h	4 h	24 h
P0	0%	6.66%	23.52%	29.01%	23.33%	17.19%
PC_5_100	74.67%	90.67%	95.56%	99.21%	96.58%	100.00%
PV_5_100	72.00%	14.67%	25.93%	52.63%	55.26%	97.37%
PC_10_100	89.00%	100.00%	100.00%	100.00%	100.00%	100.00%
PC_5_150	44.67%	1.33%	3.70%	5.26%	42.11%	94.21%
PV_5_150	66.67%	20.00%	12.96%	53.95%	65.79%	95.53%
PC_10_150	81.33%	100.00%	100.00%	100.00%	100.00%	100.00%
PV_5_200	40.00%	53.33%	37.04%	52.63%	44.74%	91.84%
PC_10_200	84.67%	100.00%	100.00%	100.00%	100.00%	100.00%

**Table 2:** Nanofiber/nanoparticle-covered samples.

Solution	Nanoparticles (wt.%)	Speed of Core Yarn (m/min)	Abbreviation
11% PVB dissolved in acetic acid	5% CuO	100 ± 15	PC_5_100
	5% V_2_O_5_		PV_5_100
	10% CuO		PC_10_100
	5% CuO	150 ± 15	PC_5_150
	5% V_2_O_5_		PV_5_150
	10% CuO		PC_10_150
	5% V_2_O_5_	200 ± 15	PV_5_200
	10% CuO		PC_10_200

## Abbreviations

NPs	Nanoparticles
CuO	Copper oxide
V_2_O_5_	vanadium oxide
PVB	Polyvinyl butyral
*E. coli*	Escherichia coli
CFU	Colony forming unit
LB	Lysogeny broth
ROS	reactive oxygen species
